# V/Q SPECT for the Assessment of Regional Lung Function: Generation of Normal Mean and Standard Deviation 3-D Maps

**DOI:** 10.3389/fmed.2020.00143

**Published:** 2020-04-28

**Authors:** David Bourhis, Philippe Robin, Marine Essayan, Ronan Abgral, Solène Querellou, Cécile Tromeur, Pierre-Yves Salaun, Pierre-Yves Le Roux

**Affiliations:** ^1^Service de Médecine Nucléaire, Centre Hospitalier Régional Universitaire de Brest, Brest, France; ^2^EA3878 GETBO, Université de Bretagne Occidentale, Brest, France; ^3^Service de Pneumologie, Centre Hospitalier Régional Universitaire de Brest, Brest, France

**Keywords:** V/Q SPECT, lung function, registration, Z-score, quantification

## Abstract

**Background:** V/Q SPECT/CT is attractive for regional lung function assessment, but accurate delineation and quantification of functional lung volumes remains a challenge. Physiological intra and inter patient non-uniformity of V/Q SPECT images make conventional delineation methods of functional lung volumes inaccurate. In that context it would be of interest to build statistical maps of normal V/Q SPECT to assess the physiological variability of radiotracers. The aim of this study was to generate normal mean and standard deviation maps of regional lung function as assessed with V/Q SPECT/CT, with (AC) and without (NoAC) attenuation correction.

**Methods:** During a 13 month period, 73 consecutive patients referred for suspected acute pulmonary embolism, that had normal V/Q SPECT/CT based on the interpretation of 2 independent nuclear medicine physicians, were selected. Four set of images were reconstructed: perfusion and ventilation images, AC, and NoAC, respectively. Statistical maps were created as follows: all cases were registered to a reference scan using the CT data, first with a rigid then with a non-rigid method. SPECTs reconstructions were then co-registered and normalized, and mean and standard deviation voxel-wise maps were calculated. To assess the consistency of generated maps to lung physiology and the potential impact of non-rigid registration, visual analysis and quantitative comparison with non-registered data were performed in the whole series. Quantitative comparison was also conducted in two randomly sampled independent subsets.

**Results:** Perfusion mean maps showed a continuous negative posterior to anterior gradient, majored on the AC mean map. Perfusion standard deviation maps showed higher variability in the periphery of the lungs, but especially in the posterior areas. The ventilation mean map showed a slightly positive posterior to anterior gradient on NoAC mean ventilation map, while the AC mean map showed no gradient. The NoAC ventilation SD map showed a higher variability in the periphery of the lungs as compared with AC SD map. No statistical difference in the posterior to anterior gradient measurements was found between the generated mean statistical maps and the non-registered data, either in the whole series or across the two independent datasets.

**Conclusion:** We proposed a methodology to create statistical normal maps for V/Q SPECTs. Maps were consistent with the known physiological non-uniformity and showed the impact of attenuation correction on the posterior to anterior gradient. These maps could be used for a Z-score analysis, and a better segmentation of healthy uptake areas.

## Introduction

The management of patients with lung disease is mainly based on pulmonary function tests (PFTs), which provide information about global lung function (1). However, PFTs do not provide spatial information about regional lung function, and the heterogeneity of which is well-known (2). Establishing a functional map of the regional ventilation and perfusion in the lungs may be highly relevant in many clinical situations, including pre surgical evaluation of lung cancer patients before surgery, radiotherapy planning to maximize dose to the tumor while minimizing the dose to the surrounding lungs (3), assessment of chronic obstructive pulmonary disease (4), or pre-surgical evaluation of patients undergoing lung volume reduction surgery.

The principle underlying Ventilation/Perfusion (V/Q) scintigraphy is very attractive for lung function assessment as it allows to assess and compare the regional distribution of the two major determinants of gas exchange in the lungs (5). To assess ventilation, inert gases or radio-labeled aerosols can be administrated. To assess the local pulmonary blood flow, ^99m^Tc labeled albumin macro-aggregates can be administrated (6–10), so that they can be trapped in terminal pulmonary capillaries. The volume concentration activity is then proportional to regional perfusion function.

Although V/Q scintigraphy is a very attractive test for regional lung function assessment, the test has not been widely accepted in clinical practice. The main explanation is that accurate delineation and quantification of functional lung volumes remains a challenge (5, 11). The transition from planar imaging to SPECT and more recently SPECT/CT has improved the quantitative capability of the test (12). 3D images avoid overlaps and thus, enable high contrast imaging and higher precision of uptakes localization and quantification. However, regional lung function delineation and quantification remains challenging for several reasons. First, although increasingly available, an absolute quantification of SPECT images remains complex, especially for V/Q imaging, as it is impossible to measure precisely the amount of radioactivity inhaled for ventilation images. It is then difficult to differentiate the counts coming from ventilation or perfusion images on perfusion SPECT. Accordingly, a relative to whole lung counts ratio is commonly used (11, 13, 14). Second, the 3D distribution of radio-tracers is physiologically not uniformly distributed into the lungs. Because of gravity, a physiological posterior to anterior gradient of varying importance can be observed when acquiring images in the supine position. Furthermore, aerosols can be trapped in the bronchi and create hot spots that can enhance heterogeneity in the SPECT images. Third, respiratory motions, whose amplitude can vary across patients, can cause artifacts in the images. Finally, SPECT-CT reconstruction parameters may also influence image delineation and quantification. Much more than Compton scattering or collimator blur corrections, attenuation correction may impact V/Q regional quantification. Published data on regional lung function quantification with SPECT commonly used attenuation corrected SPECTs (15). On the other hand, attenuation correction can be a source of image artifacts, because free-breathed acquisition can lead to unsynchronized data between CT and SPECT images (16, 17). Furthermore, clinical guidelines do not recommend the use of attenuation correction for lung imaging (7).

Because of these various factors, there is intra and inter patients physiological heterogeneity of V/Q SPECT images that make conventional delineation methods, e.g., relative segmentation methods (11), inaccurate for regional lung function assessment. In that context, it would be of interest to build statistical maps of normal V/Q SPECT to assess the variability of physiological distribution of radiotracers. This may allow better understanding of regional lung function and be used for regional lung function assessment using statistical-based delineation methods (18–20). The aim of this study was to generate normal mean and standard deviation maps of regional lung function as assessed with V/Q SPECT/CT, with and without attenuation correction.

## Materials and Methods

### Study Population

During a 13 months period, consecutive patients referred to the department of nuclear medicine of Brest University Hospital, France, for suspected acute pulmonary embolism, who had normal perfusion and ventilation scans, and no parenchyma or pleural abnormality on low dose CT based on the initial report from the nuclear medicine physician were selected. Patients with history of pulmonary disease were excluded, including patients with chronic obstruction pulmonary disease, previous pulmonary embolism, surgery or radiotherapy of the lungs. A second nuclear medicine physician reviewed all pre-selected V/Q SPECT/CT scans to confirm that all ventilation, perfusion and CT images were strictly normal. The study was approved by the institutional ethics committee (study number 29BRC19.0129).

### SPECT-CT Acquisition and Reconstruction

Dual energy V/Q SPECT-CT were performed with a continuous administration of ^81m^Krypton gas for ventilation scan (Kryptoscan® generator, Curium™, Paris, France), 5 min after intra venous administration of approximately 140 MBq of macroaggregates of albumin (MAA) labeled with ^99m^Tc (Pulmocis®, curium™, Paris, France) for perfusion scan (7). Acquisitions were performed on a Symbia Intevo 16 and Symbia T6 systems (Siemens™, Erlangen, Germany) equipped with a medium energy collimator in tomographic mode (128^2^ matrix, 128 projections of 10s), patients in feet first supine position. Energy windows were [109.9, 129.5], [129.5, 150.5], [150.7, 177.6], [177.6, 206.4] for ^99m^Tc scatter, ^99m^Tc photopeak, ^81m^Kr scatter, and ^81m^Kr photopeak, respectively. In addition, a free breathing low dose CT was performed at 130 kVp, 16 mAs with automatic exposure, pitch 1. SPECT reconstructions were performed with OSEM3D algorithm with collimator blur and Compton scatter correction, with (AC) and without (NoAC) attenuation correction. Parameters were 8 subsets 4 iterations and 8 mm Gaussian filter with 4.8 × 4.8 × 4.8 mm voxels. CT reconstructions were performed with B80 filter and 0.98 × 0.98 × 1.2 mm voxels.

### Mean and Standard Deviation Map Generation

**Figure 1:**
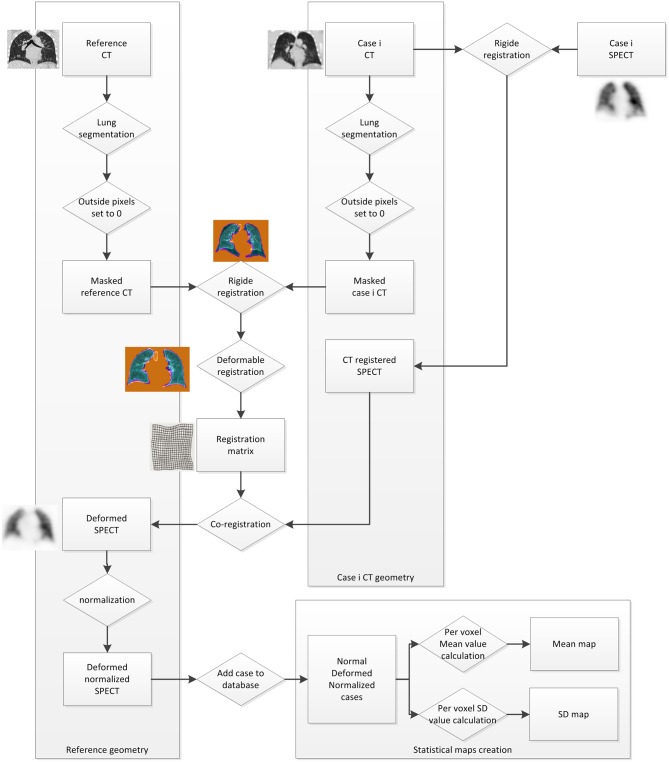
Statistical maps creation workflow.

Figure 1 describes the statistical maps creation workflow.

In order to register all cases to a single geometry, one case was selected as a reference. A case whose volume was close to the median lung volume was defined as a reference CT. All other cases were then registered to it using MiM Software (MiM v6.9, Cleveland, USA) and the VoxAlign Deformation Engine, a constrained, intensity based free-form deformable registration algorithm (21). To optimize the accuracy of the registration, lungs were segmented on CT, and all outside pixels were set to 0. Then, a rigid box-based registration between CTs was performed before the deformable image registration (21, 22). The registration matrix was saved and used to run a co-registration on both ventilation and perfusion SPECT images.

All registered examinations were then normalized using an ImageJ code (ImageJ. US National Institutes of Health, Bethesda, MD, USA). Assuming that all activity is constrained into the lungs, the normalization was based on mean value, so that it was not sensitive to hot spots. For each case, using reference CT lung segmentation, the whole lungs mean pixel value was stored for registered perfusion and ventilation scans. Then all pixels were multiplied by 1,000 and divided by the stored mean value so that the mean pixel value for all SPECTS was equal to 1,000.

Finally, from all co-registered normalized SPECT, a mean and a standard deviation value were calculated at each voxel coordinate as follows:

$$\begin{array}{l} I_{mean}\left(x,y,z\right)=\frac{1}{n}\overset{n}{\underset{i=1}{\sum }}I_{i}\left(x,y,z\right);\;\\ I_{stdev}\left(x,y,z\right)=\sqrt{\frac{1}{n-1}\overset{n}{\underset{i=1}{\sum }}{\left[I_{i}\left(x,y,z\right)-I_{mean}\left(x,y,z\right)\right]}^{2}} \end{array}$$

Where *n* is the number of database cases, *I*_*mean*_ is the mean of the pixel value from the *i* co-registered normalized cases at the *x, y, z* coordinates, and *I*_*stdev*_ the standard deviation (Figure 1). Eight 3D volumes are built from those values (Perfusion and ventilation mean and standard deviation maps, with and without attenuation correction).

### Data Analysis

Several analyses were performed in order to assess the consistency of generated maps. First, a descriptive analysis of statistical maps was performed. Second, a regional quantitative analysis was performed. Using the reference CT for lobar segmentation, relative lobar lung function (total counts in a lobe/total counts in the whole lungs) was computed. Finally, in order to assess the potential impact of deformable registration on generated 3-D maps, quantitative measurement of posterior to anterior gradient and inferior to superior gradient on generated statistical maps were compared to global parameters (mean and standard deviation) of non-registered database reconstructions. Four regions of interests were positioned on anterior, posterior, superior and inferior aspects of the right lung and mean values were recorded. Posterior to Anterior relative difference (PArd) and inferior to superior relative difference (ISrd) were calculated as follows: PArd = (Anterior-Posterior)/Posterior (%) and ISrd = (Superior-Inferior)/Inferior (%). Normal distribution was tested thanks to a Kolmogorov-Smirnov test. Mean PArd and ISrd from non-registered database were compared to the PArd and ISrd measured on generated statistical maps using a student *t*-test. Data analysis was first performed on the whole dataset.

In order to conduct the comparison across two independent datasets, two randomly sampled independent subsets of data were generated. The first subset randomly included 37 of the 73 clinical V/Q SPECT datasets which were used to produce new mean and SD maps. The second subset included the remaining 36 collected clinical V/Q SPECT datasets. The 2 subsets were independent as there were composed of different unrelated V/Q SPECT scans. In order to assess the potential impact of deformable registration on generated 3-D maps across these two independent datasets, quantitative measurement of gradients on generated statistical maps were compared to global parameters (mean and standard deviation) of non-registered database reconstructions. Using the same methodology as for the whole series, four regions of interests were positioned on anterior, posterior, superior, and inferior aspects of the right lung and mean values were recorded. PArd and ISrd were calculated. Mean PArd and ISrd from the individual subset of non-registered SPECT images were compared to the PArd and ISrd measured on generated statistical maps using a student *t*-test. Statistical analysis was performed thanks to XLSTAT software (addinsoft).

### Z-Score

In order to demonstrate the ability to calculate a Z-score map from the statistical maps, the method was tested in a V/Q SPECT/CT scan of a patient with acute PE. The voxel-wise Z-score calculation followed similar registration methodology as used for database creation. Database CT was registered to the clinical case CT, and the registration applied on database mean and SD images. Normalization was performed on the clinical case SPECT according to the mean value, measured using CT lung segmentation. Finally, Z-score map was calculated at each voxel coordinate as follows:

$$\begin{array}{l} {Z-score}_{patient}\left(x,y,z\right)\\ =\frac{\left[Pixelvalue\left(x,y,z\right)-MEANmapValue\left(x,y,z\right)\right]}{SDmapValue\left(x,y,z\right)}; \end{array}$$

## Results

### Population

From the first of January 2016 to the 11th of November 2017, 502 V/Q SPECT/CT scans were performed. Out of them, 96 had a normal V/Q SPECT/CT based on the initial report. Twenty-two were excluded after the second interpretation by an independent nuclear medicine physician and one had unusable data. Seventy-three cases were therefore included. The mean age was 59 years ±20 and 46 (60%) were female. Administrated activity of 99mTc-MAA was 155 ± 18 MBq.

### Visual Analysis

**Figure 2:**
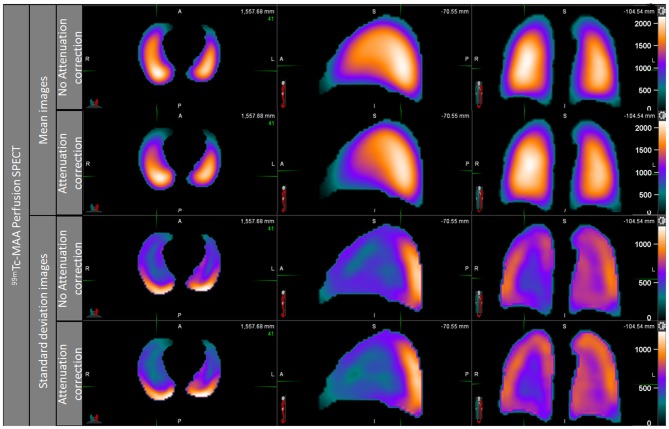
Perfusion mean map and standard deviation map, noAC, and AC.

NoAC and AC perfusion statistical maps are shown in Figure 2. Images showed a continuous negative posterior to anterior gradient, majored on the AC mean map. The perfusion standard deviation (SD) maps showed higher variability in the periphery of the lungs, but especially in the posterior areas of the lungs. This high variability in posterior area was equivalent on AC and NoAC images.

**Figure 3:**
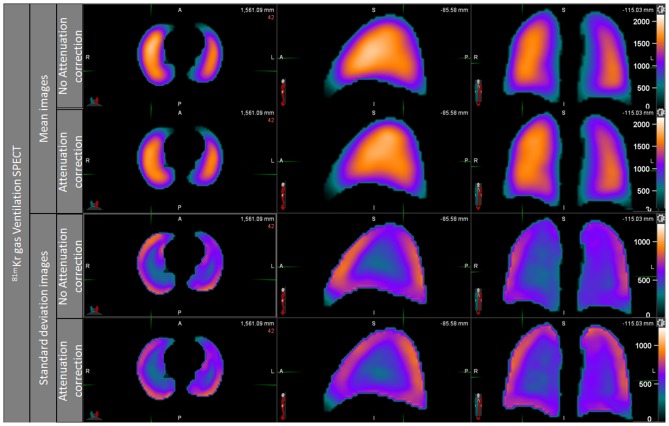
Ventilation mean map and standard deviation map, noAC, and AC.

The ventilation mean map showed a slightly positive posterior to anterior gradient on NoAC mean ventilation map, while the visual observation of AC ventilation mean map showed no gradient (Figure 3). The NoAC ventilation SD map showed a higher variability in the periphery of the lungs. The higher variability was found in the anterior area of the noAC map.

### Quantitative Analysis

Relative lobar lung function computed on the generated AC and NoAC maps are presented in Table 1. Calculation was performed on left lower lobe (LLL), left upper lobe (LUL), right lower lobe (RLL), right middle lobe (RML), and right upper lobe (RUL). The differences between NoAC and AC maps were lower than 3% for perfusion and 2.3% for ventilation in all lobes.

**Table 1:** Regional quantitative analysis of perfusion and ventilation mean map, AC, and NoAC.

**Contour**	**LLL**	**LUL**	**RLL**	**RML**	**RUL**
Perfusion (NoAC)	20.20%	24.20%	23.80%	9.70%	22.2%
Perfusion (AC)	21.6%	22.9%	25.9%	8%	21.6%
Ventilation (NoAC)	16.70%	26.30%	20.30%	12.00%	24.70%
Ventilation (AC)	17.6%	24.8%	22.6%	11%	23.9%

**Figure 4:**
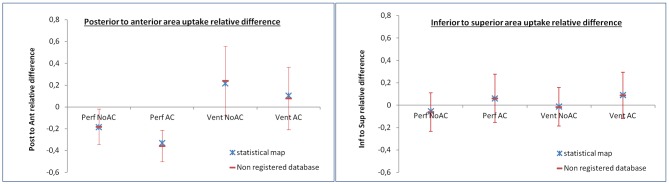
PArd and ISrd results. Mean and standard deviation of PArd and ISrd of the 73 individual scans are displayed in red. The PArd and ISrd values measured on the statistical mean maps are displayed in blue.

Mean and standard deviation of PArd and ISrd on individual data, along with the PArd and ISrd value measured on generated maps are presented in Table 2 and Figure 4. Kolmogorov-Smirnov test confirmed normal distribution. Considering posterior to anterior gradient, on perfusion generated maps, PArd was −18.3% on NoAC reconstructions, and −32.9% on AC reconstructions, respectively. On ventilation generated maps, PArd was 21.8% on NoAC reconstructions, and 10.4% on AC reconstructions, respectively. Considering inferior to superior gradient, on perfusion generated maps, ISrd was −5.4% on NoAC reconstructions, and 6% on AC reconstructions, respectively. On ventilation generated maps, ISrd was −1% on NoAC reconstructions, and 9.1% on AC reconstructions, respectively. No statistical difference was found between non-registered database and the statistical maps.

**Table 2:** Posterior to anterior and inferior to superior relative differences: mean and standard deviation on individual data vs. measured value on statistical mean map.

**POST to ANT relative difference whole dataset**		**Individual data mean (*SD*) (*n* = 73)**	**Mean map measure (*n* = 73)**	***p*-value**
Perfusion	NoAC	−18.1% (16.2)	−18.3%	0.91
	AC	−35.8% (14.4)	−32.9%	0.09
Ventilation	NoAC	24.1% (31.7)	21.8%	0.54
	AC	7.9% (28.7)	10.4%	0.46
**INF to SUP relative difference whole dataset**		**Individual data mean (*****SD*****) (*****n*** **=** **73)**	**Mean map measure (*****n*** **=** **73)**	***p*****-value**
Perfusion	NoAC	−6.1% (17.2)	−5.4%	0.73
	AC	6.3% (21.6)	6%	0.94
Ventilation	NoAC	−1.5% (17.2)	−1%	0.81
	AC	8.8% (20.6)	9.1%	0.92

The results of the second analysis on two randomly sampled independent subsets are presented in Table 3. No significant difference was found between non-registered database and the statistical maps.

**Table 3:** Posterior to anterior and inferior to superior relative differences: mean and standard deviation on non-registered vs. measured value on statistical mean map on two randomly sampled independent subsets.

**POST to ANT relative difference independent subsets**		**Individual data mean (*SD*) (*n* = 36)**	**Mean map measure (*n* = 37)**	***p*-value**
Perfusion	NoAC	−21.4% (17.1)	−20.8%	0.83
	AC	−39.7% (13.7)	−35.1%	0.05
Ventilation	NoAC	27.7% (31.2)	19.3%	0.12
	AC	10.9% (30.9)	8.8%	0.69
**INF to SUP relative difference independent subsets**		**Individual data mean (*****SD*****) (*****n*** **=** **36)**	**Mean map measure (*****n*** **=** **37)**	***p*****-value**
Perfusion	NoAC	−6.7% (15.7)	−2.7%	0.13
	AC	4.8% (24.1)	6.8%	0.63
Ventilation	NoAC	2.2% (18.4)	−0.9%	0.31
	AC	12.3% (24.6)	6.8%	0.15

### Z-Score

**Figure 5:**
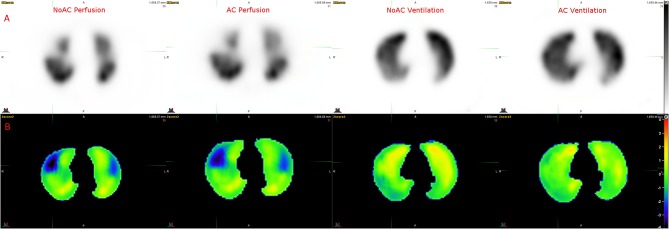
Example of Z score analysis. **(A)** Axial slices of NoAC Perfusion, AC Perfusion, NoAC Ventilation and AC Ventilation. **(B)** The corresponding generated Z-score maps.

Figure 5 shows the Z-score maps calculated using a V/Q SPECT/CT scan with acute PE. Ventilation Z-score map did not show significant variation, consistent with visual interpretation. Perfusion Z-score map showed clear low Z-score areas (<−1 SD) in the pulmonary regions with PE.

## Discussion

In this study, we generated normal statistical ventilation and perfusion SPECT/CT maps, with and without AC, based on 73 normal ventilation/perfusion lung-SPECTs. Perfusion images showed a continuous posterior to anterior negative gradient, with high variability in the posterior aspect of the lungs. Ventilation images demonstrated mild continuous positive posterior to anterior gradient, with increased variability on the periphery of the lungs. Visual and quantitative analyses showed the consistency of generated maps with lung physiology and previously published data.

Perfusion maps showed continuous posterior to anterior negative gradient. This gradient effect has been observed in multiple studies (23–27) and is explained by the influence of gravity and patient's position on pulmonary blood flow. If the perfusion variability was increased in the periphery of the lungs, probably as a consequence of registration process and respiratory motions, the variability was much higher in the posterior areas of lungs, reflecting the variability of the physiological posterior to anterior gradient across patients (27). On the other hand, we found no significant gradient in the inferior to superior direction, probably because ^99m^Tc-MAA is administrated in supine position, and patients never stand up between administration and SPECT acquisition. Gravity, and thus patient's positioning appeared to be the major factor influencing physiological regional lung uptake variability.

Ventilation mean maps showed a more unformed distribution. ^81m^Kr is an inert gas and is distributed evenly throughout the lungs. We observed a positive posterior to anterior gradient. This can also be explained by gravity. In opposition with perfusion, where blood and vessels were drained to the posterior zone, bronchial alveoli are crushed in the posterior zone (25) and free in the anterior area, and thus it is easier for ^81m^Krypton gas to fill the anterior cells.

Assessment of AC maps showed that the correction decreased the uptake in the anterior areas, and increased the uptake in the posterior areas of the lungs. As a consequence, AC increased the negative posterior to anterior gradient on perfusion images (from −18.1 to −35.8%) and decreased the positive posterior to anterior gradient on ventilation images (from 24.1 to 7.9%). This can be attributed to the lower Hounsfield units in the anterior areas compared to the posterior areas, because of the higher density at the posterior region of the lungs (crushed bronchial alveoli). Although free-breathed acquisition may potentially lead to misregistration between CT and SPECT images, we did not observed increased uptake variability in the inferior areas of the lungs on AC images. Accordingly, although clinical guidelines do not recommend the use of AC for lung images (10), our results suggest that AC images may be more relevant from a quantification perspective.

To create a statistical map of normal lung V/Q SPECT, an elastic registration was performed on all images. This technique has been employed for cardiac or brain SPECT studies (28–30) to obtain statistical regional quantification (Z scores). The registration algorithm is a free-form intensity based registration method, using a vector field between the two images (optical flow) (31). We choose to perform registration using the CT component rather than the SPECT data because we wanted this methodology to be efficient in patients with pathological SPECTs, i.e., with lung territories with no uptake. If a pathological SPECT is registered to a normal one with elastic registration, low uptake areas would be erased, while CT registration based on anatomical components will maintain those areas in quite the same proportions.

All registered reconstructions had then to be normalized. Normalization was performed on the basis of mean lungs value. The mean value was chosen for two reasons. First, this avoids normalization on hot spots. It is indeed common to find hotspots on lung SPECTs, especially on ventilation scans in the proximal bronchi, but also on perfusion scans. Second, almost all radiotracer is contained in the lungs. All ^99m^Tc-MAA are stopped in pulmonary micro circulation, when a very low quantity of free ^99m^Tc can be disseminated in the patient's body. For ventilation scan, 81mKr gas can be found only in the lungs, bronchi and trachea, in the condition that it is not spitted out because of a bad respiratory system. Thus, normalization can be very effective for lung SPECT (32).

Our results illustrate the complexity of delineating regional lung function on SPECT imaging. Lung V/Q SPECT segmentation is not trivial because of regional variability associated with the low spatial resolution. Multiple methods have been proposed like thresholds methods, local threshold methods, histogram-based threshold methods, but all have difficulties to deal with physiological heterogeneity (3, 33, 34). A Z-score based method (Z-score threshold) could help to discriminate normal and abnormal uptakes, taking into account statistical variability, as shown in Figure 5.

Our study has some limitations. First, we used 81mKrypton gas for ventilation images. Our results could therefore not be extrapolated to V/Q SPECT scans performed with ^99m^Tc labeled aerosols. Krypton is preferred for several reasons. It has a better distribution in lungs, do not create hotspots, and has lower impact on perfusion uptake. A generation of statistical lung maps using ^99m^Tc labeled aerosols would be of value. Second, the methodology required deformable registration, which may affect SPECTs regional uptake. However, quantitative analysis showed consistent results between non-registered SPECTs and statistical mean maps, either on the whole dataset or on two randomly sampled independent subsets. Relative lobar lung function quantification was also consistent with previously published data (15). Accordingly, the workflow process did not much affect SPECTs regional uptake. Furthermore, in order to compare pathological cases to the generated statistical maps, similar deformable registration will be performed.

## Conclusion

We proposed a methodology to create statistical normal maps for V/Q SPECTs. Those maps are consistent with the known physiological heterogeneity of regional lung function. Generated maps could be used for a Z-score analysis, and could lead to a better segmentation of healthy uptake areas, taking into account the physiological heterogeneity of lung images and thus improve the quantification of regional lung function.

## Data Availability Statement

The datasets generated for this study are available on request to the corresponding author.

## Ethics Statement

The studies involving human participants were reviewed and approved by Ethics Committee University Hospital Brest. The patients/participants provided their written informed consent to participate in this study. Written informed consent was obtained from the individual(s) for the publication of any potentially identifiable images or data included in this article.

## Author Contributions

DB, ME, P-YL, and P-YS contributed to designing the study. DB, CT, ME, PR, P-YL, P-YS, RA, and SQ contributed to managing imaging procedures. DB, PR, P-YL, and P-YS contributed to analyzing the data. All authors contributed to writing the manuscript, read, and approved the final manuscript.

## Conflict of Interest

The authors declare that the research was conducted in the absence of any commercial or financial relationships that could be construed as a potential conflict of interest.
